# Elastin-like polypeptide coacervates as reversibly triggerable compartments for synthetic cells

**DOI:** 10.1038/s42004-024-01270-8

**Published:** 2024-09-04

**Authors:** Chang Chen, Ketan A. Ganar, Robbert J. de Haas, Nele Jarnot, Erwin Hogeveen, Renko de Vries, Siddharth Deshpande

**Affiliations:** Laboratory of Physical Chemistry and Soft Matter, Wageningen University and Research, Stippeneng 4, 6708 WE Wageningen, The Netherlands

**Keywords:** Biophysical chemistry, Peptides, Self-assembly, Microfluidics, Synthetic biology

## Abstract

Compartmentalization is a vital aspect of living cells to orchestrate intracellular processes. In a similar vein, constructing dynamic and responsive sub-compartments is key to synthetic cell engineering. In recent years, liquid-liquid phase separation via coacervation has offered an innovative avenue for creating membraneless organelles (MOs) within artificial cells. Here, we present a lab-on-a-chip system to reversibly trigger peptide-based coacervates within cell-mimicking confinements. We use double emulsion droplets (DEs) as our synthetic cell containers while pH-responsive elastin-like polypeptides (ELPs) act as the coacervate system. We first present a high-throughput microfluidic DE production enabling efficient encapsulation of the ELPs. The DEs are then harvested to perform multiple MO formation-dissolution cycles using pH as well as temperature variation. For controlled long-term visualization and modulation of the external environment, we developed an integrated microfluidic device for trapping and environmental stimulation of DEs, with negligible mechanical force, and demonstrated a proof-of-principle osmolyte-based triggering to induce multiple MO formation-dissolution cycles. In conclusion, our work showcases the use of DEs and ELPs in designing membraneless reversible compartmentalization within synthetic cells via physicochemical triggers. Additionally, presented on-chip platform can be applied over a wide range of phase separation and vesicle systems for applications in synthetic cells and beyond.

## Abstract (web summary)

Compartmentalization within living cells is vital to orchestrate intracellular processes, but effective compartmentalization and organization within synthetic cells remains a key challenge. Here, the authors report a lab-on-a-chip system to reversibly trigger the formation of peptide-based coacervates as membraneless organelles via pH/temperature/osmolyte variations within cell-mimicking confinements.

## Introduction

Compartmentalization is essential to life, as evidenced by the universality of biological cells being enclosed within a lipid membrane. Cells exhibit further sub-compartmentalization to regulate and communicate between thousands of interconnected biochemical reactions taking place within the cytoplasm. Along with well-known membrane-bound compartments, phase-separated membrane-free compartments formed via liquid-liquid phase separation (LLPS), have been identified in recent years to play an equally important role in cellular processes^1–3^. These membraneless organelles (MOs) regulate the intracellular organization and biochemistry through sequestration of molecules, acting as reaction centers, and providing organizational hubs^4–9^.

Synthetic cells are engineered, cell-inspired systems aimed at simulating various aspects of biological cells using basic cellular building blocks^10^. Constructing such entities leads to insights into the fundamental biological principles of cells and development of biotechnological applications^11^. One of the key advantages of synthetic cells is that they do not necessarily require the exact materials used by natural cells but can instead draw inspiration from its biological counterparts^12^. This allows for synthetic molecules or modified biological macromolecules to contribute to creating synthetic cells, thus enhancing and broadening the functionality of this artificial living system.

A common approach in designing synthetic cells is to start with a biomimicking container and further encapsulate the necessary biomolecules in order to performing a specific function, with the ultimate aim of producing an autonomous, self-sufficient entity. Over the years, a variety of containers have been explored, from traditional emulsion-based containers to newer types without a clear biological counterpart^13^ such as proteinosomes^14^ and actinosomes^15^. When it comes to emulsion-based systems, water-in-oil-in-water double emulsion droplets (DEs) can be seen as an excellent intermediary between single emulsions (water-in-oil droplets) and liposomes (aqueous confinements with lipid bilayer as the boundary). In fact, many microfluidic liposome formation techniques use DEs as the starting template to ultimately form liposomes^16–18^. Compared to single emulsions which have oil as the continuous medium, DEs have a clear advantage as they are dispersed in an aqueous phase and thus have a much better access to the environment and can be easily subjected to various external biological triggers. While liposomes have a cell-mimicking lipid bilayer that is strictly needed when membrane interactions are a necessary part of the design^19–21^, DEs is a beneficial choice when the main goal is to form biocompatible, cell-sized containers in a high-throughput manner. Moreover, liposomes are relatively fragile containers owing to their flexible membrane, and are prone to shape changes^22,23^ and bursting in response to external forces^24^, and need sophisticated techniques to minimize the variation (size, lamellarity, encapsulation, etc.) within the population^25,26^. DEs on the other hand, can be much more robust against a variety of physicochemical factors including temperature, pH, and osmolytes.

Microfluidic technology can be leveraged for synthetic cell production and manipulation. From the perspective of DE production, microfluidics facilitates high production frequency, encapsulation of stoichiometrically defined complex mixtures, and size control over the droplets^27–29^. The on-chip experiments also significantly reduce the amount of encapsulated material required, making them particularly advantageous for research on synthetic cells that necessitate the use of valuable natural or synthetic materials. In terms of analyzing synthetic cell behaviors, on-chip manipulations such as sorting^30–32^ or trapping^33^ can enhance post-production control and allow for in situ visualization. Furthermore, microfluidics excels in dynamically controlling the microenvironment^34–36^, offering the potential for precise stimulation of synthetic cells.

Effective compartmentalization and organization of synthetic cells remains a key challenge^37^. Analogous to natural cells, a primary membranous vesicle with secondary sub-compartments in the form of MOs is a promising approach^38^. Low-complexity polypeptides that undergo simple coacervation are emerging as important model systems to understand and design protein-based condensates. One prominent example is that of elastin-like polypeptides (ELPs), synthetic peptides composed of VPGXG (valine-proline-glycine-guest residue-glycine) pentapeptide repeats where with the guest residue (X) can be any amino acid except proline^39^. ELPs exhibit lower critical solution temperature (LCST) and are well known for their inverse transition phase behavior, where they undergo coacervation above a transition temperature largely mediated by hydrophobic interactions^40–42^. ELPs are starting to play an important role in engineering synthetic cells, to design and program ELP-based membraneless organelles (EMOs) within cell-mimicking containers, and to achieve sub-compartmentalization^43,44^. Thus, showing a triggerable and reversible phase separation of ELP-based coacervates in cell-sized containers can be highly useful to form compartmentalized synthetic cell containers. Recently, we designed a family of pH-responsive ELPs, by tuning the fractions of charged and hydrophobic guest residues, that make them undergo LLPS in response to pH as well as temperature^45^. We further indicated their potential for forming EMOs in DEs in response to external pH trigger^45^. However, reversible and cyclic EMO formation in response to diverse physicochemical triggers in synthetic cell models remains to be explored.

In this study, we utilize DE droplets as synthetic cell containers and ELPs as components for membraneless organelles. By encapsulating our newly designed pH-responsive ELPs^45^, we examined EMO formation-dissolution cycles within DEs under various physicochemical triggers. We began the on-chip DE production allowing excellent encapsulation of the ELPs. We first demonstrated pH-triggered EMO formation within DEs, followed by dissolution, controlled via external pH. We then explored temperature-based EMO cycling, significantly reducing the time required for the EMO cycle. To gain further control, we developed a microfluidic platform integrating a dial-a-wave junction to feed in chemical triggers, and hydrodynamic traps to confine the DEs with negligible mechanical effects from the external fluid flow. Using this setup, we demonstrated multiple EMO formation-dissolution cycles in trapped DEs over a course of several hours, triggered via osmotic changes. In summary, we show pH/temperature/osmolyte-triggered EMO formation-dissolution cycles within cell-sized DEs. The microfluidic platform we have developed additionally demonstrates significant potential for broader applications in studying phase separation cycles of other molecules within micro-confinements.

## Results

### Lab-on-a-chip settings allow high-throughput production of DEs and efficient encapsulation of ELPs

**Fig. 1:**
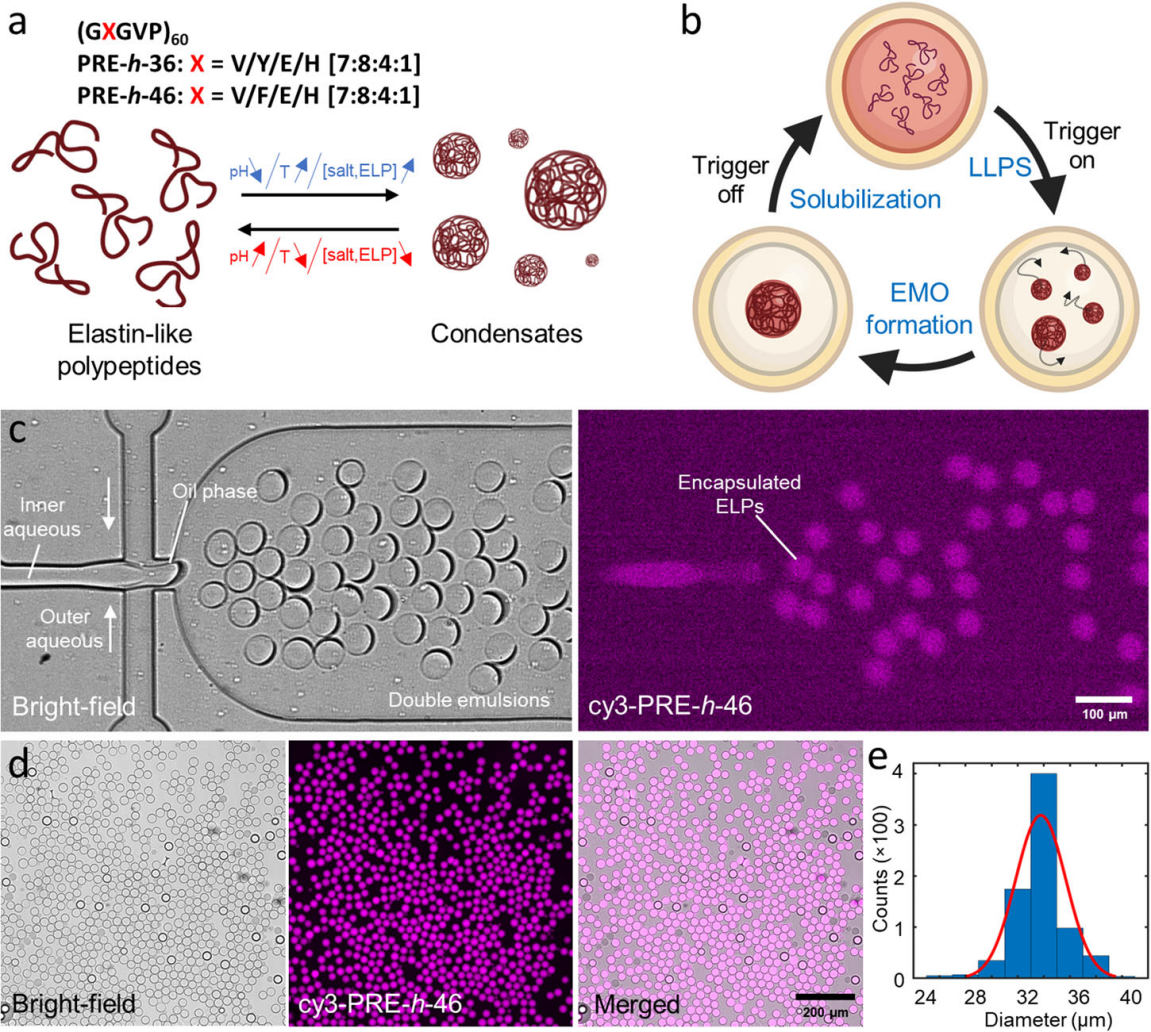
On-chip production of double emulsions (DEs) as synthetic cell containers, encapsulating elastin-like polypeptides (ELPs) as precursors for membraneless organelles. **a** We used ELPs that undergo reversible LLPS on pH decrease, temperature increase, as well as increase in the salt and protein concentrations. **b** Schematic showing the DE as a synthetic cell container encapsulating ELPs in their soluble state. An external trigger (pH, temperature, or osmotic change) leads to coacervation, forming ELP condensates that fuse with each other to form a single EMO. Reversing the external trigger leads to coacervate dissolution and the DE is restored to its initial homogenous state. **c** Bright-field and fluorescence images of the second junction showing DE production and efficient encapsulation of ELPs. **d** Bright-field, fluorescence, and a merged field-of-view showing a uniform DE population. A few dark droplets are unwanted single emulsions formed as a byproduct of the production process. **e** Frequency histogram showing the size distribution of the formed DEs (*n* = 767 from a single production batch). The red line indicates a Gaussian fit to the distribution. The inner aqueous phase contained 25 µM PRE-*h*-46 (with 4 mol% cy3-PRE-*h*-46 for fluorescence visualization).

Owing to their responsiveness to multiple triggers (pH, temperature, salt and protein concentrations), we used our previously designed pH-responsive ELPs^45^ for forming condensates (Fig. 1a). We used two variants (see Fig. 1a): PRE-*h*-46 ((GXGVP)_60_, with X = V/F/E/H [7:8:4:1]; transition pH of 5.1 at 21 °C, and transition temperature of $\approx$58 °C at pH 7.4) and PRE-*h*-36 ((GXGVP)_60_, with X = V/Y/E/H [7:8:4:1]; transition pH of 5.7 at 21 °C, and transition temperature of $\approx \,$45 °C at pH 7.4)^45^. PRE-*h*-46 is thus suitable to study pH-triggered coacervation while PRE-*h*-36 is more amenable to temperature-triggered coacervation. Our idea was to use their reversible LLPS behavior to form dynamic and responsive EMOs within DEs. Figure 1b shows the concept pictorially: A synthetic cell (DE) encapsulating the ELPs remains homogenous in absence of trigger. A pH/temperature/osmolyte-based external trigger induces LLPS of ELPs inside the synthetic cell, and the formed ELP coacervates ultimately coalesce together to form a single EMO freely diffusing in the lumen. Switching off the trigger leads to the dissolution of the EMO, making the interior homogenous again. This EMO formation-dissolution cycle can be repeated on demand to compartmentalize the vesicle interior as per the need.

To obtain efficient encapsulation of the ELPs in DEs in a high-throughput manner, we produced the DEs in a lab-on-a-chip setting. We used standard soft-lithography and microfabrication to prepare polydimethylsiloxane (PDMS)-based microfluidic devices (see Materials and Methods for details; device design is provided as Supplementary Data 1). The inner aqueous phase gets encapsulated in the DEs, the organic phase is a fluorinated oil with surfactants (FluoSurf-C) and forms the shell-like boundary, and the outer aqueous phase is a buffered solution to match the osmolarity of the inner aqueous phase and keep the DEs stable. Figure 1c shows the second junction of a typical production (also see Supplementary Movies 1 and 2): the inner aqueous containing the solution of interest (containing cy3-labeled ELP in this case to facilitate fluorescence visualization) is pinched off at the first junction (not shown) to first form single emulsions which subsequently get transferred into the outer aqueous buffer to form DEs. We operated the device at a production rate between 100–500 Hz, thus obtaining $\sim$10^6^ DEs in a typical 1 h of production. The formed DEs showed excellent encapsulation of the inner aqueous, as can be seen from the high fluorescent intensity within the DEs and very low background signal (Fig. 1d). The produced DE population was also monodispersed; Fig. 1e shows the size distribution of DEs produced in a single batch with the mean diameter of 33.8 µm ± 1.92 µm (mean ± standard deviation; coefficient of variation <6%; *n* = 767). The produced DEs were collected and stored at 4 °C where they remained stable over weeks.

### Reversible triggering of EMOs via pH changes

**Fig. 2:**
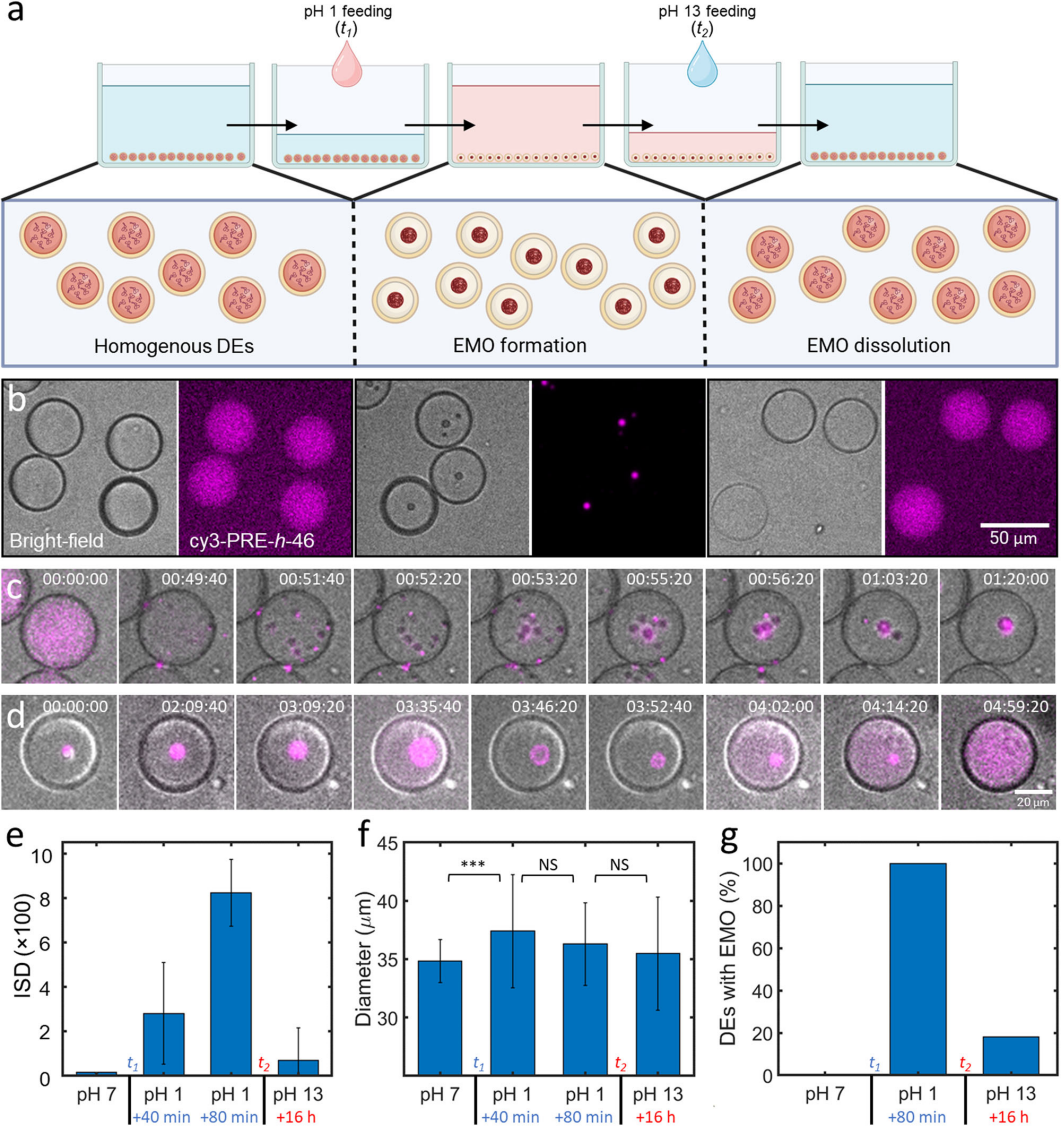
Reversible pH-triggered EMO formation in DEs. **a** Schematic of the experimental cycle induced by pH changes leading to EMO formation and dissolution within a PDMS well; *t*_*1*_ and *t*_*2*_ correspond to the time points of adding pH 1 feed and pH 13 feed respectively. **b** Representative bright-field and fluorescence images of DEs across three conditions: pre-pH shock (left), pH 1 bath (middle), and pH 13 bath (right). **c**, **d** Time-lapse (bright-field and fluorescence merged) of EMO formation (**c**) and dissolution (**d**) within a single DE monitored over >6 h. The contrast has been adjusted for individual images for better visualization. **e** Mean ISD values of the DE population at different time points over a period of 20 h showing clear correlation with EMO formation and dissolution. **f** Mean diameter of DEs at different trigger points showing a significant difference (marked by asterisks; *p*-value < 0.001) after adding the acidic feed, while no significant difference (marked NS) was observed later on. **g** A plot showing the percentage of DEs containing EMOs after different triggers. Data points represent mean values derived from at least 83 DEs for each measurement, with error bars indicating standard deviations. The inner aqueous phase contained 25 µM PRE-*h*-46 (with 4 mol% cy3-PRE-*h*-46 for fluorescence visualization).

Upon optimizing the DE production and ELP encapsulation, we first tested the pH-triggered EMO cycle using PRE-*h*-46. Figure 2a shows a schematic of the experimental procedure employed to trigger the EMO cycle, where we put the ELP-containing DEs in a PDMS-based well to perform microscopic observations, covering the top with a glass slide to prevent evaporation (see Materials and Methods for details). Figure 2b provides the corresponding bright-field and fluorescence micrographs showing the key steps. Initially, the ELPs remained homogenous within the DEs when immersed in an aqueous solution at pH 7.4 (left panels). We initiated EMO formation by introducing a “switch-on” trigger by simply substituting majority of the bulk buffer volume ($\approx$92%) with an acidic solution (pH 1). The low pH environment led to a gradual acidification of the interior of DEs, and within 80 min, each DE developed a single compartment exhibiting intense fluorescence, marking EMO formation (middle panels). We then introduced the second “switch-off” trigger, by substituting majority of the external acidic solution ($\approx$92%) with an alkaline solution (pH 13). This reverted the acidic lumen of the DEs back towards a neutral pH and initiated the EMO dissolution process. Within 16 h, 82% of the DEs (*n* = 111) again showed a homogenous interior, indicating a successful EMO formation-dissolution cycle (right panels). Figure 2c shows the time-lapse for a single DE responding to the acidic environment, demonstrating the start of LLPS, droplet coalescence, and ultimately EMO formation. Interestingly, after the alkaline feeding, we occasionally observed an expansion of EMO into a ring-like structure, followed by gradual dissolution and finally complete homogenization to the initial state (see Fig. 2d). We analyzed the EMO cycle based on the variation in the fluorescence within each DEs interior, in the form of intensity standard deviation (ISD), as can be seen in Fig. 2e. The initial homogenous DEs had a relatively low variation in the intensity in their interior and thus a low value of ISD, while the ISD value increased notably upon EMO formation, as the ELPs got enriched in the dense coacervate phase, equilibrated with a dilute phase with a very weak fluorescence. Subsequent EMO dissolution again resulted in a uniform fluorescence intensity across the DEs, and thus lower ISD values.

We would like to note that to ensure that the EMO formation was triggered solely by pH, a hypotonic acidic solution was used (see Materials and Methods for details). This resulted in a significant increase in the size of DEs within the first 40 min, as can be seen in Fig. 2f (*p*-value < 0.001) but the DEs remained stable, without any bursting. This increased DE volume ($\sim$23% increase) decreased the concentration of the encapsulated salts and ELPs, both factors resisting LLPS^41,46^. Thus, the observed EMO formation, in spite of salt and ELP dilution, confirms pH as the LLPS trigger. Even after 16 h of alkaline feeding, 18% of the population (*n* = 111) still contained EMOs (see Fig. 2g). This observed resistance is likely not caused by evaporation-induced hypertonic shock, leading to DE dehydration and internal concentration increase, because the evaporation was minimized by covering the well with the cover slide. Indeed, the size of DEs did not change significantly after adding the alkaline solution, even after 16 h (see Fig. 2f). The very low permeability of the oil shell to water and thus a large equilibration time, especially compared to the experimental time, could instead be responsible for the observed slow dissolution process and not all DEs attaining complete dissolution (see Fig. 2d). We also carried out two consecutive EMO formation-dissolution cycles (Supplementary Fig. 1), albeit with a lower efficiency for the second cycle for reasons mentioned above. Additionally, we followed the dissolution of EMOs in the first cycle over a period of 20 h (Supplementary Fig. 2). EMO dissolution was much slower for the overall population compared to their formation, and the percentage of DEs containing EMOs decreased sharply after 7.5 h, likely corresponding with the increase of the internal pH above the transition pH. In conclusion, this pH-triggered cycle demonstrated the possibility of realizing EMO formation and dissolution inside DEs upon chemical changes in the external buffer.

### Reversible triggering of EMOs via temperature changes

**Fig. 3:**
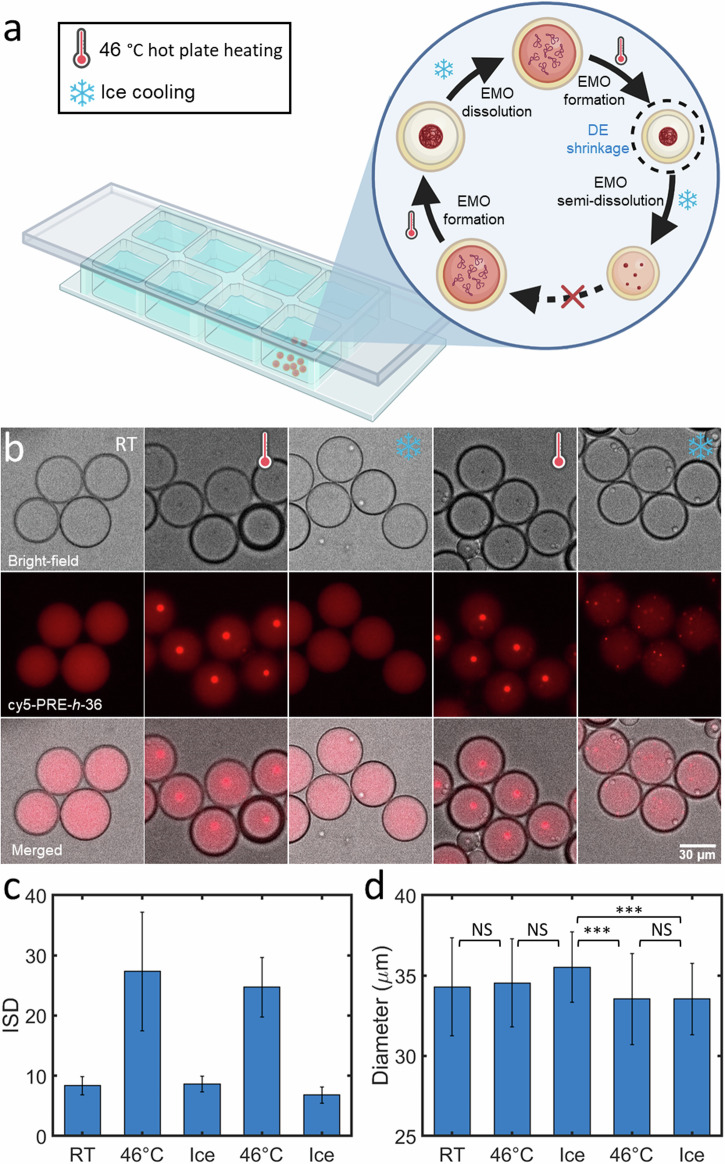
Reversible temperature-triggered EMO formation in DEs. **a** Illustrative representation of the cycle induced by temperature changes leading to EMO formation and dissolution within a closed PDMS well. **b** Representative bright-field, fluorescence, and merged images of several DEs showing two temperature-triggered EMO formation-dissolution cycles: pre-heating (first panel), post-heating at 46 °C for 20 min (second and fourth panels), and after cooling on ice for 15 min (third and fifth panels). **c** Mean ISD values of the DEs at the corresponding trigger points. **d** Corresponding mean diameters of the DE population showing significant reduction in size after the second heating (marked by asterisks; *p*-value < 0.001), while no significant difference (marked NS) was observed otherwise. Data points represent mean values derived from >39 DEs, with error bars indicating standard deviations. The inner aqueous phase contained 25 µM PRE-*h*-36 (with 4 mol% cy5-PRE-*h*-36 for fluorescence visualization).

In order to capitalize on the responsiveness of our ELPs also to temperature, we probed whether temperature cycling would lead to a more rapid EMO formation. We utilized PRE-*h*-36 due to its more amenable transition temperature of 45 °C at pH 7.4 compared to PRE-*h*-46^45^. Figure 3a depicts a schematic of the experimental setup used to initiate the EMO cycle via temperature changes. Similar to the previous experiment, we placed the ELP-containing DEs in a PDMS-based well for microscopic observations, while a cover glass slide was placed on top to prevent evaporation. Figure 3b displays the corresponding bright-field and fluorescence micrographs detailing five steps of the process. Initially, at room temperature, the ELPs were homogeneously distributed within the DEs (first panels). We triggered EMO formation by placing the PDMS device on a hot plate at 46 °C for 20 min, after which each DE acquired a single EMO amidst weak background fluorescence (second panels). Subsequent cooling on ice for 15 min resulted in complete EMO dissolution (third panels), completing an EMO cycle within 35 min. We could successfully repeat the EMO formation using the same sample (fourth panels) by reheating for another 20 min at 46 °C. In the subsequent EMO dissolution, however, the DEs did not revert back to a completely homogenous fluorescence state but showed tiny residual ELP coacervates after cooling on ice for 15 min (fifth panels). The ISD values for the corresponding five panels are shown in Fig. 3c and showed similar trend for the entire DE population. Nonetheless, for the last cooling, the mean ISD value was similar to that of after the first cooling, indicating that the majority of EMO fraction was dissolved. This tendency to not completely dissolve in the second cycle could be resulting from DE shrinkage, elevating the internal ELP and salt concentrations, due to increased evaporation rates during the heating cycle. Size analysis confirmed a significant decrease (*p*-value < 0.001) in the mean DE diameter ($\approx$5.6%) after the second heating cycle (Fig. 3d). In conclusion, this temperature-triggered experiment demonstrated rapid EMO formation and dissolution across multiple cycles, although cycle repeatability was constrained by evaporation induced by temperature changes. These off-chip pH- and temperature-triggering showed us the possibility to develop ELP formation-dissolution cycles within DEs but at the same time also exhibited the limitations when handling the DEs in bulk settings. This is why we turned our attention to developing an on-chip triggering and observation system.

### Dial-a-wave junction and microfluidic traps enable controlled environmental changes combined with long-term localization and visualization of DEs

**Fig. 4:**
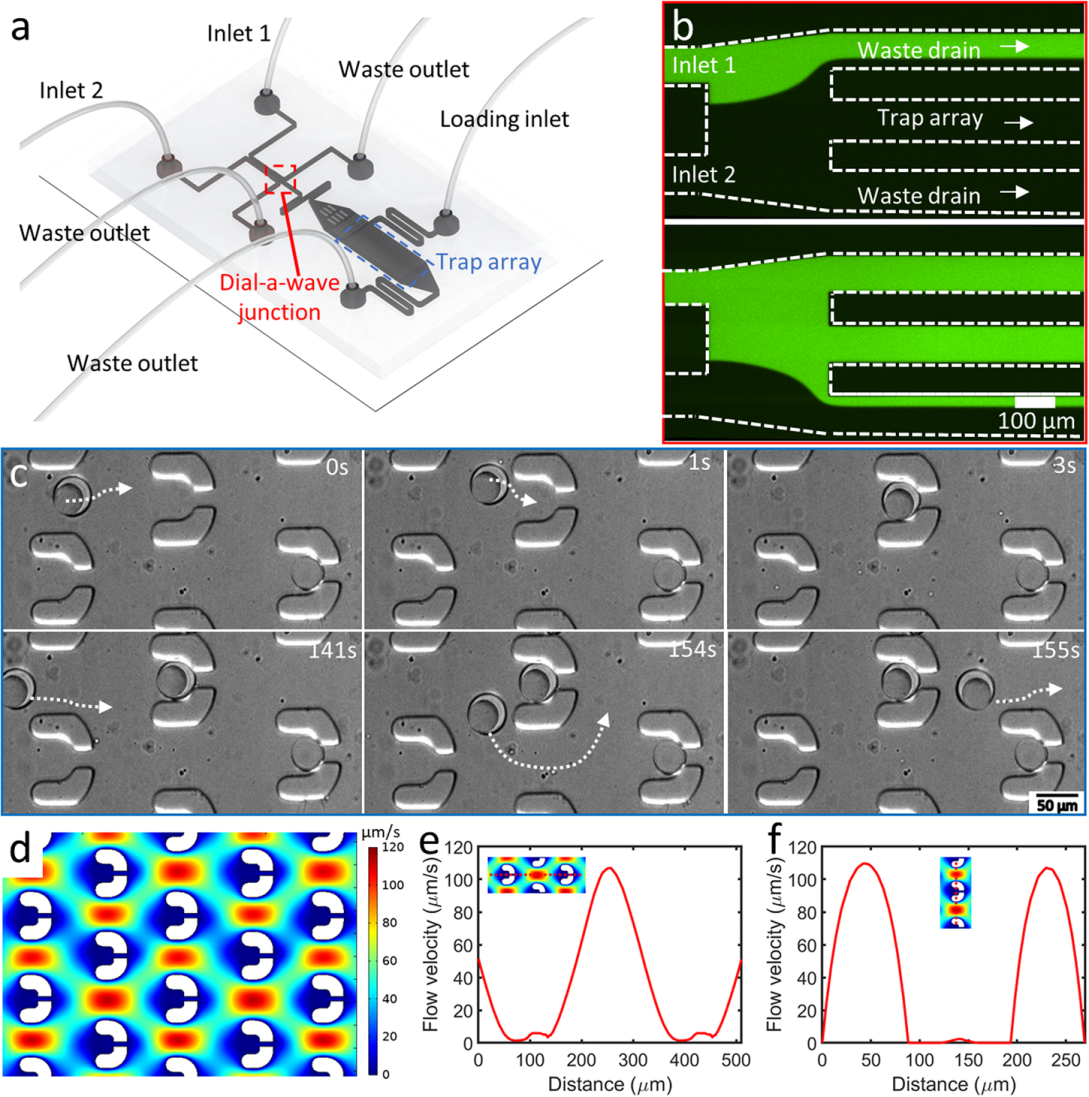
DEs can be trapped on chip and subjected to continuous modulation of the external environment. **a** Diagram illustrating the microfluidic device, which incorporates a dial-a-wave (DAW) junction for selective feeding and U-shaped hydrodynamic traps for DE containment. **b** Working principle of the DAW junction. The top image depicts a ‘switch-off’ trigger when higher pressure is applied at inlet 2 (PBS), while the bottom image depicts the “switch-on” trigger allowing the feed from inlet 1 (containing 10 µM FITC in PBS) to flow into the main channel containing the traps. **c** Trapping process for DEs, with the top panel showing a DE entering and being confined within the trap, whereas the bottom panel demonstrates that once a trap is filled, the incoming DE bypasses the filled trap. White arrows indicate the DE trajectories. **d**–**f** Computational simulation results for the fluid flow in microfluidic chip. **d** The fluid velocity cloud chart within the trap array. **e** Fluid velocity distribution along the longitudinal axis of the traps. **f** Fluid velocity distribution along the vertical axis of the traps.

After demonstrating the EMO cycle in bulk settings, we sought for an on-chip system that would allow us to visualize the DEs over a prolonged period and trigger multiple cycles effectively without disturbing the DEs. We thus designed a second chip in which we could flow in the produced DEs and visualize the EMO formation in a controlled fashion. The idea of the device is sketched in Fig. 4a (device design is provided as Supplementary Data 2), and it consists of two modules. A dial-a-wave (DAW) junction to enable us to switch the external environment between two triggers and an array of physical traps to immobilize the DEs. The DAW junction is based on a previous study^34^, and has two feeding ports to allow for fast switching between the two inputs. These two channels meet at a junction bifurcating into three outlets. The middle outlet feeds into the trap arrays, while the outer two outlets function as waste channels and allow the flow in the middle outlet to vary between carrying one of the two inputs, without getting any backflow into the inactive inlet. As can be seen in Fig. 4b, the DAW junction was able to provide a switch on/off function by simply adjusting the pressure ratio of the two feeding ports. A fluorescent solution (10 µM fluorescein isothiocyanate (FITC) in phosphate buffer saline (PBS)) and a non-fluorescent solution (PBS) was used for the demo (also see Supplementary Movie 3, and Materials and Methods for details). DAW junction was operated at low pressures (<60 mbar), with a switch time in the order of seconds.

Downstream the DAW junction, we designed a trap array based on previous designs^33^ and the traps were scaled to fit the DEs with a size of $\approx$40 µm. Each trap had a narrow (10 µm) opening in the rear to allow the external solution to pass through but not the trapped DE. Figure 4c shows a typical trapping sequence of DEs. Due to the narrow gap at the tail of the U-shaped trap, DEs tended to flow in and get trapped in it. Trapped DE significantly increased the flow resistance within the trap and restricted the flow across it. As a result, filled traps redirected the fluid flow to the gaps between the other traps and avoided further interaction of incoming DEs with trapped ones. The remaining DEs then got localized in the traps downstream or simply exited the chip. We further carried out finite element analysis to demonstrate the fluid flow around the traps (see Materials and Methods for details). As can be seen in Fig. 4d, the staggered trap array results in significantly low-velocity regions inside the traps. Figure 4e indicates a nearly linear decrease in flow velocity from 106.9 – 1.4 µm/s from the trap periphery to the interior. Similarly, while the flow velocity on the sides of the traps reached 109.6 µm/s, the fluid velocity in the center of the traps is only 2.4 µm/s (see Fig. 4f). Thus, within these traps, flow-induced mechanical force subjected on the localized DEs is ignorable, and the external stimuli are basically in the form of chemical changes obtained via the DAW junction. It should be noted that we also observed non-specific immobilization of DEs outside the traps but these DEs also remained stable and responded to the triggers in a similar fashion as that of the trapped DEs. While further optimization is needed to avoid such non-specific immobilization and maximize the trapping efficiency, these instances also confirm that the traps do not affect the behavior of DEs in any way except fixing their spatial orientation.

### Reversible triggering of EMOs via osmolarity changes

**Fig. 5:**
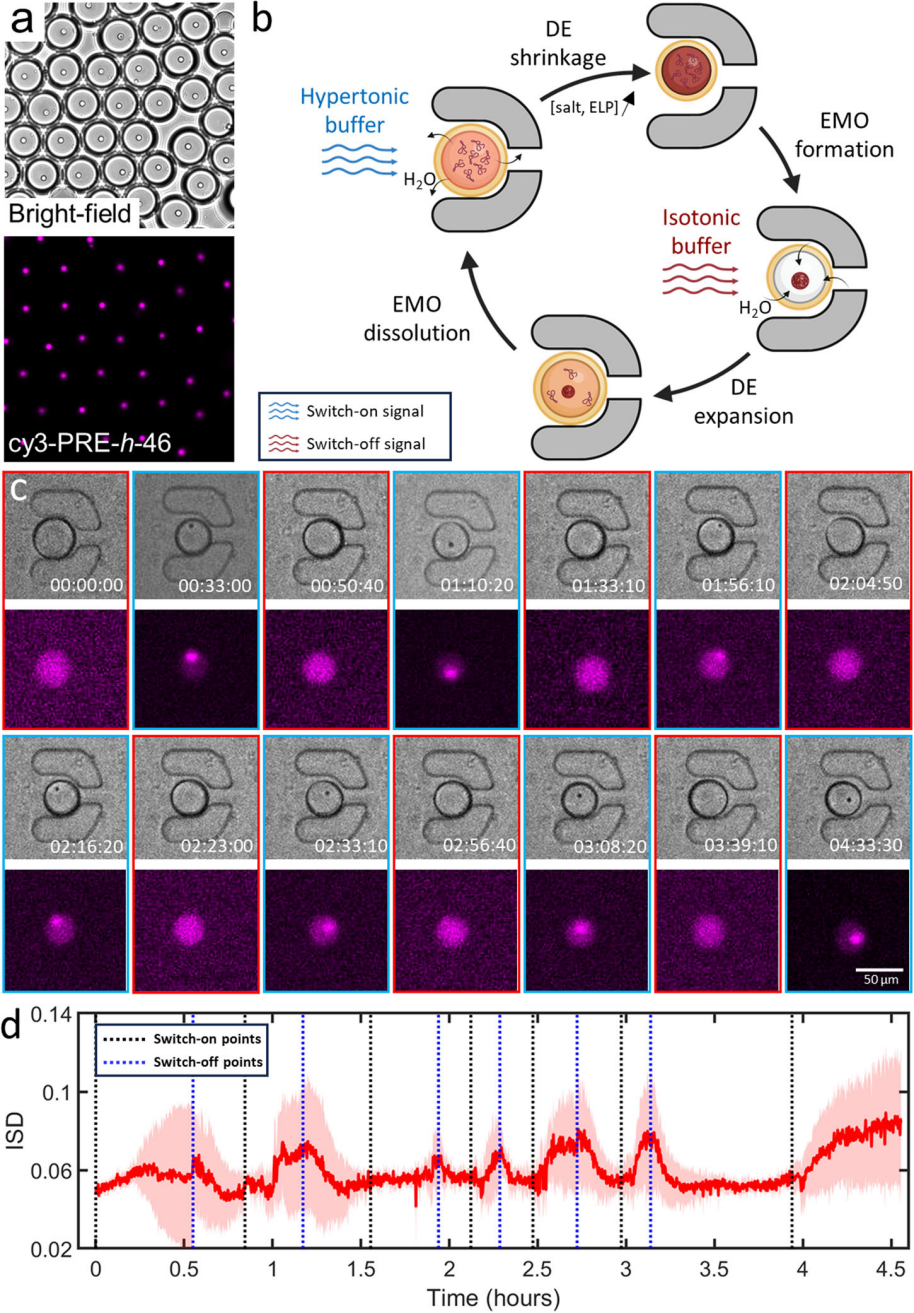
Changes in osmotic strength of the external environment leads to reversible EMO formation in DEs trapped on-chip. **a** Triggering coacervation via osmolarity change led to a single, uniform-sized EMO in each DE as visualized in both bright-field and fluorescence. **b** Illustrative representation of the cycle induced by concentration changes leading to EMO formation and dissolution within microfluidics. The process involves entrapment of the DE, followed by a hypertonic shock, leading to DE shrinkage and subsequent EMO formation as a result of increase in salts and ELP concentration. Subsequent introduction of an isotonic solution reverses this process, thereby dissolving the EMO. **c** Time-lapse bright-field and fluorescence images showing the dynamic process of EMO formation and dissolution in response to repeated hyperosmotic shocks within a trapped DE. **d** Change of the mean ISD of DEs (n $\ge$ 13) over a period of 4.5 h. The pink shaded area indicates the standard deviation of the ISD values. Black and blue dotted lines show approximate introduction times of the hypertonic and isotonic solutions respectively. The inner aqueous phase contained 25 µM PRE-*h*-46 (with 4 mol% cy3-PRE-*h*-46 for fluorescence visualization).

With the production and manipulation chips in operation, we set out to reversibly trigger EMOs in DEs encapsulating PRE-*h*-46. To demonstrate the EMO formation in response to osmolarity change, we first performed a bulk experiment by putting a droplet containing DEs on a glass slide and letting the outer aqueous phase evaporate, leading to a hypertonic external solution. This resulted in an efflux of water from the DEs to balance the osmolarity, and as a consequence, increased the salt and ELP concentration in their lumen. Since the increased concentration of salts (such as phosphates^46^ and sodium chloride^47^) and ELPs^41,48–50^ both lower the transition temperature of ELPs, their up-concentration indeed triggered the LLPS process, and led to EMO formation in the DE population, with each vesicle containing a single EMO (Fig. 5a). After demonstrating this bulk osmolarity-triggered EMO formation process, we switched to the on-chip setting to perform this in a more sophisticated and reversible fashion. As illustrated in Fig. 5b, the scheme consisted of introducing a hypertonic solution through one inlet to shrink the DEs via osmosis and thus trigger EMO formation. Subsequently, by switching to an isotonic solution via the other inlet, the DEs would be restored to their original size, leading to EMO dissolution. The on-chip setting would ideally allow multiple on-off triggering cycles without mechanically disturbing the trapped DEs. Figure 5c shows a time-lapse of a single trapped DE demonstrating EMO formation-dissolution seven times in a row (also see Supplementary Movie 4). The red-bordered panels depict the dissolved states of EMOs, whereas the blue-bordered panels illustrate their condensed states. The decrease in size of the DE, as observed in the bright-field panel, occurred after introducing the hypertonic solution. Concurrently, the emergence of black dots, colocalizing with intensely fluorescence areas, confirmed the EMO formation. Figure 5d reveals the dynamic changes in the ISD values during the multiple triggers across 4.5 h. We would like to note that we also took into account non-specifically immobilized, i.e., not present in the traps, DEs for the analysis as they exhibited the exact same behavior as the trapped DEs. Each switch-on operation (introduction of the hypertonic solution) led to a concomitant increase in ISD, signifying a local redistribution in the form of coacervation of ELP molecules within the DEs. Conversely, the switch-off operation (introduction of the isotonic solution) resulted in a reduction of ISD, attributed to the dissolution of EMOs. Regardless of the long- or short-time intervals between the triggers, we observed similar EMO formation-dissolution process and its manifestation into ISD values, indicating the stability of the DEs and the on-chip manipulation system. This successful execution of seven continuous cycles within 4.5 h confirms the stability of DEs within the chip and the precise control over EMO dynamics.

## Discussion

Liquid-liquid phase separation and coacervation-based biomolecular condensates are becoming increasingly important in the synthetic cell research to bring about efficient compartmentalization and form reaction hubs. In this work, we have demonstrated multiple reversible coacervation cycles of peptide-based condensates in response to various external physicochemical triggers in cell-sized double emulsion droplets using lab-on-a-chip systems. We have shown the utility of pH-responsive ELPs as model membraneless organelles for synthetic cells^45^. Due to the sequence specificity and thus unique microenvironment of each ELP, EMOs could be further developed to harbor specific chemical reactions or to design multiple distinct MOs within synthetic cells.

pH is a crucial physiological parameter within the cellular environment. Thus, the response of synthetic cells and their internal contents to pH changes is essential for simulating cellular behaviors. Prior studies have illustrated the pH-responsive complex coacervation cycle in liposomes and the capability of the resultant coacervates to serve as temporary containers for enzymatic reactions^9^. In this paper, we leveraged the pH-responsive properties of our engineered ELPs to trigger a simple coacervation cycle in DE-based synthetic cell models, thus demonstrating their potential as novel reversible membraneless organelles responding to pH stimulation. Optimizing production parameters (such as thinner oil shell or different oil composition) may allow the use of milder pH changes without significant delay in response times.

Temperature is another crucial physical factor in cellular environments, and motivated by its significance, temperature-triggered LLPS of ELPs has been extensively studied in single emulsion systems^42,51,52^. Here, we demonstrated multiple cycles of EMO formation in a double emulsion system by modulating the external temperature. Although the temperatures used were higher (>40 °C) than those typically found in natural cellular environments, we believe that optimizing the design of ELPs or other molecules for the lower transition temperature could make it feasible to achieve synthetic EMO cycles within physiological temperatures.

Along with pH and temperature, we have also demonstrated the capability to induce reversible LLPS through osmotic changes. This clearly demonstrates efficient water flux across the oil shell of DEs, driven by an osmotic pressure difference, without compromising their structural integrity. By creating hypertonic/isotonic environments, the change in ELP and salt concentration successfully triggered LLPS in the DEs^41,46^. This method can be used more universally, as the biopolymer and salt concentrations play a crucial role in the phase separation process.

Since the pioneering work in early 2000s^27^, monodisperse DEs are widely used for fundamental and applied purposes. Nevertheless, compared to the extensive use of single emulsions^53–55^, DEs have rarely been used as synthetic cell containers to study coacervate systems. While the use of double emulsions as the container have certain clear limitations, such as the lack of interactions between coacervation and the membrane^56^, our work has demonstrated the excellent stability of DEs across a wide pH range (from pH 1 till pH 13), rapid temperature changes (from 0 – 46 °C) and significant osmotic pressure differences (up to 400 mOsm), highlighting their versatility and resilience in dynamic environments. Even under drastic volume changes (up to 23%), the oil shell effectively serves as a reservoir for surfactant molecules, storing excess surfactants during DE contraction and redistributing them during expansion. The DEs-based synthetic cell model thus provides an efficient and stable confinement for various basic physicochemical triggers.

With respect to microfluidics, the DAW junction to toggle between “on” and “off” triggers provides an easy access to two distinct environments for DEs to induce multiple EMO formation-dissolution cycles. The system can be upgraded by allowing a continuous spectrum between the two triggers via mixing the streams in well-defined ratios before the solution enters the trap arrays. The post-DAW junction mixing can be enhanced using multi-height ‘herringbone’ structures^35^ or cylinder arrays^57^, enabling rapid adjustments in the feeding solution composition. The on-chip setup can prove handy in triggering LLPS cycles in response to a wide variety of chemical triggers (pH, ionic strength, protein concentration, etc.).

In conclusion, our DE- and ELP-based approach can serve as a versatile synthetic cell system, to study the formation and dissolution of biopolymer-based membraneless organelles within cell-sized confinements. In parallel, the microfluidic platform sets the stage for long-term entrapment, rapid and dynamic stimulation, and real-time observation of synthetic cell dynamics.

## Materials and methods

### Materials

Chemicals—sodium chloride, potassium chloride, disodium hydrogen phosphate and dihydrogen sodium phosphate, polyvinyl alcohol (PVA; 87–90% hydrolyzed, average MW 30–70 kDa), propylene glycol methyl ether acetate (photoresist developer), and FITC were purchased from Sigma-Aldrich. Sylgard™ 184 silicone elastomer and curing agent were purchased from Dow. Silicon wafer was bought from Silicon Materials. Photoresist (SU8-50) were purchased from micro resist technology GmbH. Microfluidic accessories including Tygon® tubing 1/16” ODx 0.02”, biopsy punchers, XXS microfluidic reservoir and HFE 7500 fluorinated oil containing 2% FluoSurf-C surfactant were purchased from Darwin Microfluidics. Elveflow pressure controller OB1-MK3 was used to control the fluid flow.

### Microfabrication and surface functionalization

Master wafers were prepared according to the previously described microfabrication method^25^ and the protocol was adjusted to attain the channel height of 20 µm. To prepare the microfluidic devices for DE production and trapping, PDMS and the curing agent (SYLGARD™184 elastomer) were mixed in 10:1 weight ratio. The mixture was poured on the master wafer and degassed using vacuum desiccator. Meanwhile, a glass coverslip (Corning® #1) was coated with PDMS by spin coating at 500 rpm for 15 s (at an increment of 100 rpm/s) and then at 1000 rpm for 30 s (at an increment of 500 rpm/s). Both PDMS-covered wafer and PDMS-coated glass were baked at 70 °C for 2 h. The hardened PDMS block was carefully removed, and inlets and outlet holes were punched using a biopsy punch of diameter 0.75 mm. The PDMS block was then bonded on the coverslip after 30 s of plasma treatment at 12 MHz (RF mode high) using a plasma cleaner (Harrick Plasma PDC-32G). The bonded device was then baked at 70 °C for 2 h.

After baking, the production chip underwent a PVA (5% w/v, molecular weight 30 −70 kDa) treatment as described previously^25^. Briefly, the outer aqueous inlet was flowed with a PVA solution whereas the inner aqueous and oil inlets were kept at a positive pressure to retain the PVA-air boundary stable at the second production junction. After 15 min of incubation, PVA solution was pushed out by applying maximum pressure (2 bar) on the inner aqueous and oil inlets and the extra liquid was removed by applying a negative pressure (−1 bar) at the exit. The device was then dried for 15  min by baking on a hot plate at 120 °C.

PDMS wells were prepared using a clean silicon wafer without any pattern. First, non-patterned PDMS blocks were prepared similarly to the description above by pouring PDMS-curing mixture over the wafer, followed by baking and removing the cured material from the wafer. The blocks were then punched for 5 mm hole puncher to make the wells. After plasma bonding on a PDMS-coated glass slide, the wells were surface-functionalized by pipetting PVA solution in them and incubating for 15 min. The PVA was pipetted out and the device was baked on a hot plate at 120 °C for 15 min. After surface functionalization, all devices were stored at room temperature.

### ELP synthesis and labeling

Briefly, the plasmid construction followed standard methods for generating recombinant proteins, including cloning of synthetic gene fragments, recursive directional ligation by plasmid reconstruction with BamHI and XhoI restriction endonucleases, Sanger sequencing, transformation into *E. coli*, and expression. After expression, the ELPs were purified using inverse transition cycling purification method, including inoculation and culture growth, induction of protein expression, cell harvesting and sonication, centrifugation (cold spin and hot spin), dialysis, and lyophilization. PRE-*h*-46 and PRE-*h*-36 were labeled by conjugating with sulfo-cy3 or sulfo-cy5 maleimide dyes respectively. Free dye was removed by multiple rounds of spin filtration. Labeled proteins were stored at −20 °C for further use. The full synthesis and labeling details can be found elsewhere^45^. In pH and concentration triggers (Figs. 2 and 5), PRE-*h*-46 and cy3-PRE-*h*-46 were used. For temperature trigger (Fig. 3), PRE-*h*-36 and cy5-PRE-*h*-36 were used. The addition of labeled ELPs, either 4 mol% cy3-PRE-*h*-46 or 4 mol% cy5-PRE-*h*-36 (24 μM unlabeled and 1 μM labeled), was to facilitate fluorescence imaging.

### Double emulsion production

Three solutions—inner aqueous (IA), outer aqueous (OA), and oil phase—were prepared for the DE production. IA and OA contained 100 mM NaCl and 50 mM Na_2_HPO_4_ at pH 7.4. IA additionally contained the appropriate ELPs while OA contained 1% v/v tween-20 surfactant. HFE 7500 fluorinated oil (containing 2% FluoSurf-C surfactant) was used as the oil phase. The three solutions were connected to respective microfluidic inlets on the device via Tygon® tubing. OA, oil phase, and IA solutions were flown in, and after making sure no air bubbles were present in the device, the pressures of the three inlets were adjusted to ensure a stable double emulsion production. In order to avoid wastage of the IA solution during the start-up phase of the DE production, Milli-Q water was used in the place of IA till stable DEs were produced. A clean pipette tip (200 µL) was inserted into the outlet to collect the produced DEs. The DE dispersion was stored in amber-colored bottles and kept at 4 °C.

### pH-trigger experiment

The pH adjustment was performed by replacing the external solution in which the DEs were dispersed. Initially, the PDMS well was filled by pipetting in 110 µL of OA and 10 µL of DE suspension. For the experiment with one cycle, 110 µL of the supernatant was removed in each exchange, and the well was immediately refilled with the respective feeding solutions. The incubation times for pH 1 and pH 13 feed were 80 min and 16 h respectively. For the experiment with two cycles, the incubation times of the DEs were set to be 20 h after all feedings, except for the first 2 h-triggering from pH 7.4 to 1. After each 20 h-incubation, we refilled the well with Milli-Q water (approximately to the same level) to eliminate the effects of evaporation. The compositions of the acidic and alkaline feeding solutions were as follows: (i) acidic (pH 1): 50 mM NaCl, 100 mM HCl, 1% tween-20; (ii) alkaline (pH 13): 50 mM NaCl, 100 mM NaOH, 1% tween-20. The well was consistently covered with a coverslip except during solution exchanges.

### Temperature-trigger experiment

Temperature adjustments were made by placing the PDMS device in different environments. Initially, the PDMS well was filled with 110 µL of OA and 10 µL of DE suspension, and was covered with a coverslip. For heating, the PDMS device was positioned on a hot plate (VWR®, VMS-D), pre-heated at 46 °C for 20 min. For cooling, the device was placed on top of an ice-filled cooler box for 15 min.

### Switching demonstration of the DAW junction

The DAW junction was operated by tuning the pressures on the two inlets. Inlet 1 contained PBS with 10 µM FITC at pH 7.4 while inlet 2 contained PBS at pH 7.4. For the switch-off state, the respective pressures applied on inlet 1 and 2 were 10 and 20 mbar. To switch on, inlet 1 pressure was increased from 10 – 40 mbar in steps of 5 mbar, and finally returned to 20 mbar.

### On-chip trapping and osmolyte-based triggering

The trapping and DAW chip was operated using three solutions: a feeding solution containing DEs and the two trigger solutions for each of the DAW inputs. Feeding solution was obtained by dispersing the produced DEs in OA. The trigger solutions to induce osmotic changes were (i) hypertonic: 100 mM NaCl, 200 mM KCl, 1% tween-20, and 50 mM Na_2_HPO_4_ at pH 7.4 and (ii) isotonic (OA): 100 mM NaCl, 1% tween-20, and 50 mM Na_2_HPO_4_ at pH 7.4.

The device was first filled with Milli-Q by submerging the entire device in a petri plate and applying vacuum in a desiccator. In this water-filled device, empty tubing was inserted in the three outlets (two waste outlets and one exit), the loading inlet, and one of the feeding inlets (inlet 2). The other feeding inlet (inlet 1) was connected with a tubing connecting OA. By applying 50 mbar on inlet 1 for 15 min, the liquid in the channels was replaced by OA. Afterwards, the loading inlet was connected to a XXS microfluidic reservoir and was prefilled with OA. The DEs were then introduced into the standing reservoir and loaded on chip by applying 20 and 80 mbar pressures on inlet 1 and loading inlet, respectively. After about 10 min of loading, the loading inlet pressure was switched off and the inlet 1 pressure was increased to 50 mbar for washing away the extra DEs. Finally, the tubing connecting inlet 2, now filled with OA, was replaced by a new one for hypertonic feed solution. For EMO formation, the pressures applied on inlet 1 and 2 were 10 and 40 mbar, respectively. For EMO dissolution, the pressures were 40 and 10 mbar on inlet 1 and inlet 2 respectively.

### Microscopy

Images for switch Demo of DAW junction, pH- and osmolarity-trigger experiments were acquired using a Nikon-Ti2-Eclipse inverted fluorescence microscope equipped with pE-300^ultra^ illumination system, a Nikon Plan F 10 x /0.3 objective, and appropriate filter sets (Semrock). In DAW junction switching experiments, samples were excited with 15% LED intensity and images were acquired at exposure of 20 ms. In DE experiments, for bright-field visualization, images were acquired at an exposure of 600 μs with 5% light intensity while for fluorescence visualization, samples were excited with 50% LED intensity and images were acquired at exposure of 50 ms. All images were acquired using a Prime BSI Express sCMOS camera.

Images for temperature-trigger experiments were acquired using a ZEISS microscope (Axio Observer 7) equipped with Light Source Colibri 5 (Type RGB-UV), a ZEISS Plan-NEOFLUAR 20 x /0.5 objective, and a 90 HE LED filter set. For bright-field visualization, images were acquired at an exposure of 10 ms with 1.5 V light intensity while for fluorescence visualization, samples were excited with 20% light intensity and images were acquired at exposure of 100 ms, using a Prime BSI Express sCMOS camera.

### Numerical study

A two-dimensional finite element model was constructed in COMSOL Multiphysics 5.6 (COMSOL Inc., Sweden) to analyze the fluid dynamics within the trap array region. The model replicated the exact dimensions of the chip design used in the experiment. The laminar flow module was utilized for the simulation. The inlet flow rate was set to 1.18 mm/s, based on a rough measurement from the microfluidic experiment, while the outlet pressure was maintained at 0 Pa with backflow suppression enabled. All wall boundaries were defined as no-slip surfaces. The mesh consisted of free triangular elements ranging from 0.401 to 34.7 µm. A stationary study mode was employed, and the results were exported for further analysis using MATLAB R2019b.

### Data analysis

ImageJ was used in case of pH- and temperature-trigger experiments. First, the DEs were confirmed from the bright-field images and a circular ROI was marked. These ROIs were then used for size and intensity analysis. Only those DEs that were non-clustered and in focus were considered for the size and intensity analysis. MATLAB R2019b was used in case of osmolarity-trigger experiments. Fluorescent images of DEs were analyzed to determine both the intensity values and size changes. DE regions based on the bright-field images were used as a mask and the circular regions with an intensity higher than the surroundings were identified as ROIs. One-way analysis of variance (ANOVA, *α* = 0.05) was employed to assess if there were significant differences in the sizes of DEs under different conditions. Error bars in the graphical representations indicate the standard deviation of the mean for each sample.

## Supplementary information

Supplementary information

Description of Additional Supplementary Files

Supplementary Data 1

Supplementary Data 2

Supplementary Data 3

Supplementary Data 4

Supplementary Movie 1

Supplementary Movie 2

Supplementary Movie 3

Supplementary Movie 4

## Data Availability

The MATLAB code used for image processing (Fig. 5) is provided as Supplementary Data 3.

## Data availability

Data supporting the findings of this study are available within the paper, its Supplementary Information, Supplementary Movie 1–4, Supplementary Data 1–4. Any additional supporting data are available from the corresponding authors upon reasonable request.
