# Supplementary material for: Elastin-like polypeptide coacervates as reversibly triggerable compartments for synthetic cells
Source: Commun Chem. 2024 Sep 4;7:198. doi: 10.1038/s42004-024-01270-8 (PMC11374812; doi:10.1038/s42004-024-01270-8)
Supplement: Supplementary file 1 — Supplementary information [file 42004_2024_1270_MOESM1_ESM.pdf]

Supplementary Information

Elastin-Like Polypeptide Coacervates as Reversibly Triggerable Compartments for Synthetic Cells

Chang Chen[1], Ketan A. Ganar[1], Robbert J. de Haas[1], Nele Jarnot[1], Erwin Hogeveen[1], Renko de Vries[1], Siddharth Deshpande[1§]

[1]Laboratory of Physical Chemistry and Soft Matter, Wageningen University and Research, Stippeneng 4, 6708 WE Wageningen, The Netherlands

§ corresponding author; email: siddharth.deshpande@wur.nl

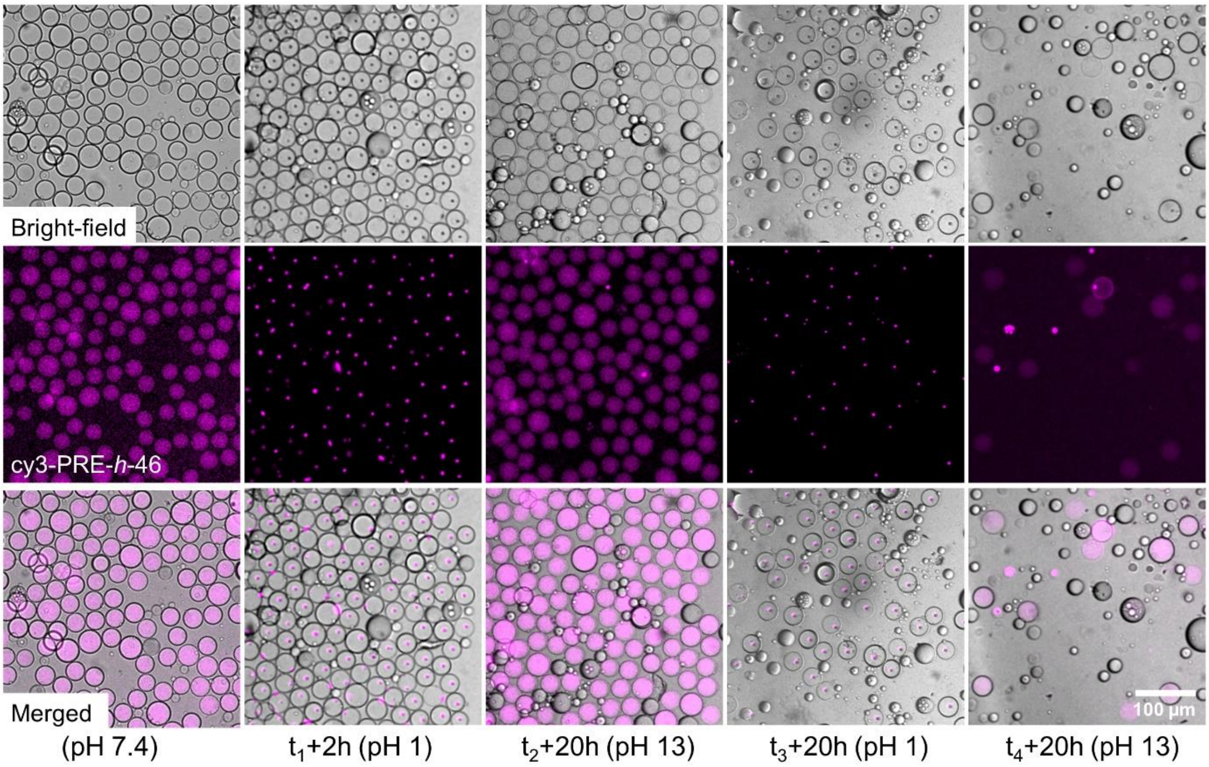

Supplementary Figure 1. pH-triggered two consecutive EMO formation-dissolution cycles within DEs. Representative bright-field, fluorescence, and merged images of several DEs showing two pH-triggered EMO formation-dissolution cycles; t_1, t_2, t_3, and t_4 correspond to the time points of adding pH 1 feed (first cycle), pH 13 feed (first cycle), pH 1 feed (second cycle), and pH 13 feed (second cycle) respectively.

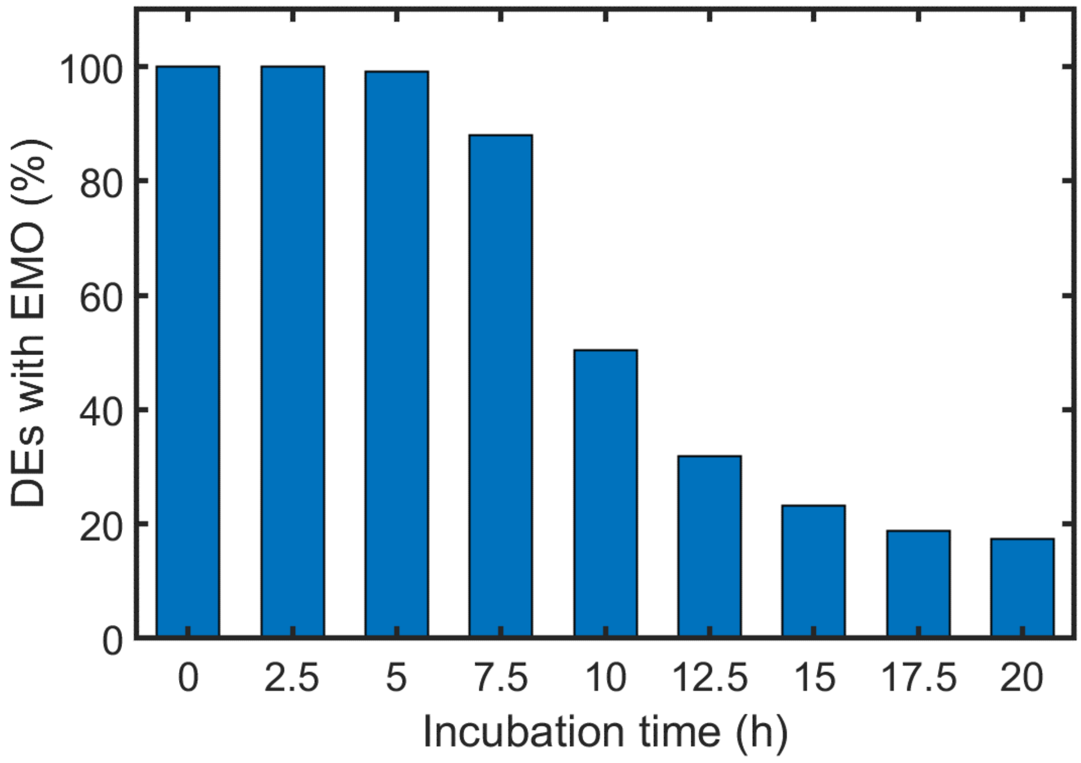

Supplementary Figure 2. pH-triggered dissolution of EMOs over time. Percentage of DEs containing EMOs at different time points upon pH 13 trigger (*n* > 300 for each time point). A steep decrease in EMOs can be seen after 7.5 hours.
