# Supplementary material for: Elastin-like polypeptide coacervates as reversibly triggerable compartments for synthetic cells
Source: Commun Chem. 2024 Sep 4;7:198. doi: 10.1038/s42004-024-01270-8 (PMC11374812; doi:10.1038/s42004-024-01270-8)
Supplement: Supplementary file 2 — Description of Additional Supplementary Files [file 42004_2024_1270_MOESM2_ESM.pdf]

Description of Additional Supplementary Files

File name: Supplementary Data 1

Description: Design file for the double emulsion production.

File name: Supplementary Data 2

Description: Design file for double emulsion trapping and stimulation.

File name: Supplementary Data 3

Description: Analysis code used for image processing.

File name: Supplementary Data 4

Description: Data underlying the figures in the main article and its supplementary information.

File name: Supplementary Movie 1

Description: Bright-field video showing on-chip high-throughput DE production.

File name: Supplementary Movie 2

Description: Fluorescence video showing efficient encapsulation of ELPs during on-chip DE production. The inner aqueous phase contained 25 µM PRE-h-46 (with 4 mol% cy3-PRE-h-46 for fluorescence visualization).

File name: Supplementary Movie 3

Description: Fluorescence video showing efficient adjustment and quick response of the DAW junction. Inlet 2 pressure was kept at 20 mbar whereas the inlet 1 pressure was increased from 10 to 40 mbar in steps of 5 mbar, and finally returned to 20 mbar.

File name: Supplementary Movie 4

Description: Bright-field (above) and fluorescence (below) channels showing four consecutive EMO formation-dissolution cycles within trapped DEs in response to repeated osmotic changes. The DEs contained 25 µM PRE-h-46 (with 4 mol% cy3-PRE-h-46 for fluorescence visualization).
